# Cerebral venous sinus stent insertion as a primary versus secondary procedure in the treatment of intracranial hypertension

**DOI:** 10.1186/2045-8118-12-S1-P46

**Published:** 2015-09-18

**Authors:** Syed N Shah, Aswin Chari, Simon D Thompson, Patricia Haylock-Vize, Jinendra Ekanayake, Edward W Dyson, Andrew R Stevens, Claudia Craven, Huan W Chan, Tarek Mostafa, Neekhil A Patel, Samir A Matloob, Ahmed K Toma, Laurence D Watkins

**Affiliations:** 1 National Hospital for Neurology and Neurosurgery (NHNN), UCLH, UK

## Introduction

Venous sinus stent insertion is being increasingly used as a primary treatment for intracranial hypertension patients (BIH). However, the value of this treatment modality is still controversial. This study looks into the difference in effectiveness of stents inserted as a primary procedure and those inserted in patients who already had cerebrospinal fluid diverting shunt in place i.e. as a secondary procedure.

## Materials and methods

A retrospective case series of patients with intracranial hypertension treated in our unit with venous sinus stent insertion. Case notes were reviewed for clinical presentation, initial response to stent insertion and follow up results. Patients in group A did not have previous shunt before insertion of stent (stent as primary procedure) while group B patients already had shunt(s) in situ (stent as secondary procedure). Stent survival time was defined as number of days from stent insertion until the next intervention due to worsening/recurrent symptoms or end of follow up period (June 2015).

## Results

In total, 44 patients underwent stent insertion between 2011 and 2015 (24 patients in group A and 20 in group B). Follow-up period was 490 ± 453 days (mean +standard deviation); group A 548 ±522 days and group B 420 ±488 days (p=0.6). The stent survival time in group A was 435 ± 453 days compared with group B: 270.95 ± 394.86 days (p= 0.03). A total of 13 patients required further surgical intervention during the follow up period, of which 4 (16%) were in group A and 9 (45%) in group B (p=0.001).

## Discussion

The results suggest that stent insertion is a relatively effective method of treatment of patients with intracranial hypertension. Lower survival rates of stents inserted as a secondary procedure could be related to the fact that shunts change the cerebrospinal fluid hydrodynamics interfering with stent function or simply due to the fact that group B patients have more aggressive form of intracranial hypertension.

